# Late-onset benefit in progressive advanced hepatocellular carcinoma with continued sorafenib therapy: a case report

**DOI:** 10.1186/1752-1947-6-38

**Published:** 2012-01-26

**Authors:** Yusuke Okuwaki, Takahide Nakazawa, Hisashi Hidaka, Akitaka Shibuya, Wasaburo Koizumi

**Affiliations:** 1 Department of Gastroenterology, Kitasato University East Hospital, 2-1-1 Asamizodai, Minami-ku, Sagamihara, Kanagawa 252-0380, Japan

**Keywords:** Hepatocellular carcinoma, Sorafenib, Late-onset response, Progressive disease, Molecular targeted drug

## Abstract

**Introduction:**

In the past, no effective systemic therapy has existed for patients with advanced hepatocellular carcinoma. Sorafenib, an oral multikinase inhibitor, has recently been shown to improve overall survival in patients with advanced hepatocellular carcinoma in two randomized, double-blinded, placebo-controlled trials. This drug has been approved as the first-line therapy for advanced hepatocellular carcinoma patients. We report an intriguing case of advanced hepatocellular carcinoma in which the patient achieved late- onset partial response by prolonged administration of sorafenib in spite of progressive disease.

**Case presentation:**

A 54-year-old Japanese man was treated with sorafenib for multiple lung metastases after surgical resection for advanced hepatocellular carcinoma accompanied by vascular invasion of the left branch of the portal vein. Although the effective diagnosis was progressive disease, almost all sites began to reduce or disappear eight months after the diagnosis of progressive disease. A dramatic reduction in alpha-fetoprotein and des-gamma-carboxy prothrombin levels was observed. The patient finally achieved partial response and his status remains unchanged.

**Conclusions:**

If tolerated, prolonged sorafenib treatment may be beneficial.

## Introduction

Hepatocellular carcinoma (HCC) is the sixth most common cause of malignancies worldwide and represents the third most common cause of cancer-related death [1]. HCC frequently recurs despite curative local control such as surgical resection or local ablation therapy and the prognosis of patients with advanced HCC including invasion to intrahepatic large vessels remains dismal [2-5]. No systemic therapies in a meta-analysis were shown to confer a survival advantage over best-supportive care [6]. Recently, sorafenib (Nexavar^®^, Bayer HealthCare Pharmaceuticals), an oral multikinase inhibitor that mainly targets Raf kinases, vascular endothelial growth factor receptors 1, 2, and 3, and platelet-derived growth factor receptor beta, has been shown to improve overall survival in patients with advanced HCC in two randomized, double-blinded, placebo-controlled trials [7,8]. This drug has been approved as the first-line therapy for these patients.

The tumor response and its clinical course observed under treatment with sorafenib are rather different from the other conventional cytotoxic agents. In fact, most of the patients who responded to sorafenib had stable disease (SD) in both of these studies [7,8], and sorafenib seldom induces the dimensional tumor shrinking usually observed with conventional cytotoxic agents. Therefore, it has been suggested that sorafenib prolongs survival by delaying disease progression.

We report the case of a patient with advanced HCC who showed an intriguing clinical course. The patient achieved late- onset partial response (PR) by continuing sorafenib in spite of the progressive disease (PD).

## Case presentation

**Figure 1:**
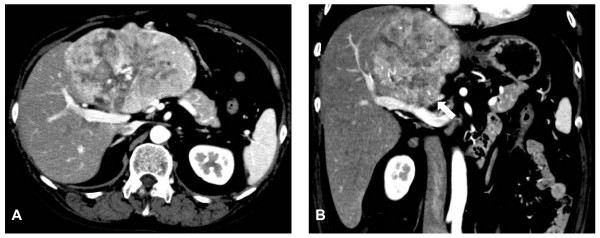
**Contrast-enhanced computed tomography scans before surgical resection**. **(A) **Arterial phase showed a large hypervascular lesion extending from the left lobe to the anterior segment of the right lobe. **(B) **The left branch of the portal vein was not identified as being involved in the tumor, invasion of the portal vein was suspicious (white arrow).

At the end of 2008, a 54-year-old Japanese man was referred to our hospital because a liver mass had been discovered by ultrasonographic examination. Enhanced computed tomography (CT) revealed a large liver tumor 15 cm in diameter extending from the left lobe to the anterior segment of the right lobe, which was enhanced in the arterial phase and washed out in the equilibrium phase, which is typical of HCC (Figure 1). The left branch of the portal vein was not identified as being involved in the tumor. Extrahepatic metastasis was not observed. The patient was in good health, and his Eastern Cooperative Oncology Group performance status was 0. He had never smoked cigarettes, drunk alcohol, or been diagnosed as obese or diabetic. Laboratory data demonstrated normal liver function and he had no evidence of past or persistent hepatitis B virus or hepatitis C virus infection. Elevations of alpha-fetoprotein (AFP) and des-gamma-carboxy prothrombin (DCP) levels were observed: AFP level 119 ng/ml (normal range < 10); DCP level 158,000 mAU/ml (normal range < 40). His Child-Pugh score was A 5, and the tumor stage was Barcelona Clinic Liver Cancer stage C. According to the clinical practice guidelines for HCC [9], surgical resection was considered because of normal underlying liver, although it was accompanied by vascular invasion. Subsequently, an extended left lobectomy was performed, and complete resection of the tumorous area was achieved. The pathological examination confirmed a moderate differentiated HCC with portal vein invasion of the left branch, and the underlying liver tissues were normal.

Eight months after the resection, recurrence of multiple lung metastases was detected on a follow-up periodical CT scan. Intrahepatic recurrence was not observed. The patient was initially treated with sorafenib at 800 mg per day. Two weeks later, a grade-2, hand-foot skin reaction occurred and a dose reduction of 400 mg per day was made. After two months, follow-up CT scans showed a growth and new metastatic lesions in the lung and liver, and PD was diagnosed according to the revised Response Evaluation Criteria in Solid Tumors Guidelines (RECIST 1.1) and modified RECIST [10,11].

**Figure 2:**
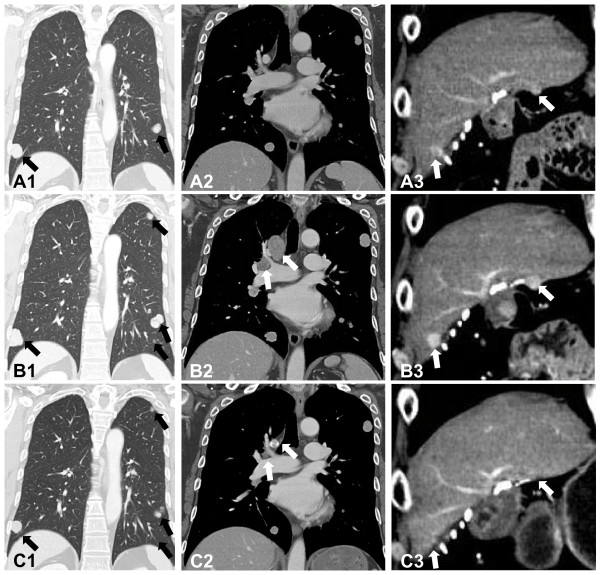
**(A1-3) Contrast-enhanced computed tomography scans before restarting sorafenib**. **(A1, 2) **Multiple metastatic lesions were observed in the lung (black arrows). **(A3) **Local tumor progressions were also observed in the cut surface of the liver (white arrows). **(B1-3) Contrast-enhanced computed tomography scans six months after restarting sorafenib**. **(B1) **The growth and new metastatic lesions were observed in the lung (black arrows). **(B2) **In addition, mediastinal lymph node metastases were observed anew (white arrows). **(B3) **Local tumor progressions were unequivocally observed (white arrows). **(C1-3) Contrast-enhanced computed tomography scans 10 months after restarting sorafenib**. **(C1) **Metastatic lesions in the lung shrunk or disappeared (black arrows). **(C2-3) **Mediastinal lymph node metastases and local tumor progressions completely disappeared (white arrows).

After the diagnosis of PD, the patient participated in a randomized, double-blind, placebo-controlled, comparative study of S-1 (TS-1, Taiho Pharmaceutical Co., Ltd., Tokyo, Japan) of an oral cytotoxic anti-cancer agent. Response evaluation after the first cycle was PD, and the treatment was discontinued. Sorafenib 400 mg per day was started again based on a consensus for the retreatment. Metastatic lesions continued to worsen over six months despite the retreatment with sorafenib (Figure 2 A1-3, B1-3); however, almost all lesions began to shrink or disappear eight months after restarting sorafenib. Dramatic reductions in AFP and DCP levels (AFP decreased from 3640 ng/ml to 263 ng/ml, and DCP from 3080 mAU/ml to 272 mAU/ml) were observed. PR was observed on the CT scan (Figure 2C1-3), and the patient remains stable on sorafenib at a dose of 400 mg per day. Currently, he is working full time and is in excellent general health.

## Discussion

Our patient achieved PR after 10 months beyond PD during long-term treatment with sorafenib. Generally, the withdrawal of molecular targeted agents including sorafenib, for a diagnosis of PD, is followed by a conventional criterion of cytotoxic anti-cancer agents. However, in the present case, treatment with sorafenib was continued based on the patient's tolerability and strong desire to continue it, and because no other second-line therapy option was available. This case showed that the manner of treatment with sorafenib, differing from the standard treatment criterion, could induce the treatment response to PR. To the best of our knowledge, this is the first report in the literature of late-onset response of sorafenib beyond PD in patients with advanced HCC. The underlying mechanism of late-onset tumor shrinkage beyond PD is unclear. One could hypothesize that the mainstream signaling pathway of the tumor involved in cell proliferation or angiogenesis might change to become more sensitive by mutation or some other alteration by prolonged administration. Retreatment with sorafenib after the first PD might be related to the late-onset response of our patient. It has been reported that tumor cell necrosis was enhanced by re-exposure to sorafenib in a study of renal cell carcinoma (RCC) xenograft models [12].

The anti-tumor response of molecular targeted agents including sorafenib seems to be different from that of the conventional cytotoxic agents because a specific response manner has been observed. For instance, approximately 70% of patients showed SD with a lower response rate (2%) in initial phase III studies of sorafenib [7]. On the other hand, rapid regression after a short period of only one or two weeks of treatment with sorafenib has been reported [13]. Several patients achieved PR after approximately 10 months of SD in patients with sorafenib for metastatic RCC [14]. These findings suggest that sorafenib could induce efficacy and clinical benefit by various methods of treatment and that molecular-targeted agents have a more complicated responsiveness than do cytotoxic agents. Therefore, we suggest that there might occasionally be responders, on long-term administration of sorafenib, among the PD cases. Further studies are required to determine factors underlying clinical outcome.

## Conclusion

If tolerated, prolonged sorafenib treatment may be beneficial. The efficacy of sorafenib on a long-term basis warrants further investigation because molecular targeted agents deliver a potent impact of various cellular signal transductions.

## Consent

Written informed consent was obtained from the patient for publication of this case report and any accompanying images. A copy of the written consent is available for review by the Editor-in-Chief of this journal.

## Competing interests

The authors declare that they have no competing interests.

## Authors' contributions

YO gathered the information for this case and was a major contributor in writing the manuscript. TN contributed in writing the Discussion and editing the manuscript. HH, AS and WK contributed to the writing of the manuscript. All authors read and approved the final version of the manuscript.
